# Nanopore sequencing technology: a new route for the fast detection of unauthorized GMO

**DOI:** 10.1038/s41598-018-26259-x

**Published:** 2018-05-21

**Authors:** Marie-Alice Fraiture, Assia Saltykova, Stefan Hoffman, Raf Winand, Dieter Deforce, Kevin Vanneste, Sigrid C. J. De Keersmaecker, Nancy H. C. Roosens

**Affiliations:** 1 Scientific Institute of Public Health (WIV-ISP), Platform of Biotechnology and Bioinformatics (PBB), J. Wytsmanstraat 14, 1050 Brussels, Belgium; 2 Scientific Institute of Public Health (WIV-ISP), Biosafety and Biotechnology Unit (SBB), J. Wytsmanstraat 14, 1050 Brussels, Belgium; 3 Ghent University (UGent), Department of Information Technology, IMEC, Internet Technology and Data Science Lab (IDLab), Technologiepark-Zwijnaarde 15, 9052 Ghent, Belgium; 4 Ghent University, Faculty of Pharmaceutical Sciences, Laboratory of Pharmaceutical Biotechnology, Ottergemsesteenweg 460, 9000 Ghent, Belgium

## Abstract

In order to strengthen the current genetically modified organism (GMO) detection system for unauthorized GMO, we have recently developed a new workflow based on DNA walking to amplify unknown sequences surrounding a known DNA region. This DNA walking is performed on transgenic elements, commonly found in GMO, that were earlier detected by real-time PCR (qPCR) screening. Previously, we have demonstrated the ability of this approach to detect unauthorized GMO via the identification of unique transgene flanking regions and the unnatural associations of elements from the transgenic cassette. In the present study, we investigate the feasibility to integrate the described workflow with the MinION Next-Generation-Sequencing (NGS). The MinION sequencing platform can provide long read-lengths and deal with heterogenic DNA libraries, allowing for rapid and efficient delivery of sequences of interest. In addition, the ability of this NGS platform to characterize unauthorized and unknown GMO without any *a priori* knowledge has been assessed.

## Introduction

The commercialization of genetically modified organisms (GMO) in the food and feed chain is controlled in several countries, such as European Union (EU), by enforcement laboratories. To this end, a real-time PCR (qPCR) based strategy is commonly used, including an initial step that allows detection of the potential presence of GMO through a qPCR screening analysis that targets elements commonly found in GMO, such as Cauliflower mosaic virus (CaMV) 35S promoter (p35S) and *Agrobacterium tumefaciens* nopaline synthase terminator (tNOS). The combination of these two elements has been reported to cover the detection of most of EU authorized GMO (~70%) and approximately 90% of EU unauthorized GMO^1–7^. Consequently, the main issue of this qPCR screening is that the presence of unauthorized GMO could potentially be concealed by the identification of authorized GMO in samples that contain both authorized and unauthorized GMO possessing the same targeted elements. A second issue is that most of the qPCR screening elements, such as p35S and tNOS, are derived from natural organisms. Therefore, their detection can only indicate the potential presence of GMO, whereas only unnatural associations of sequences can prove the actual presence of GMO^1–7^. To overcome these limitations, we have previously successfully developed an integrated strategy, combining successive qPCR screening (potential detection of GMO), DNA walking (generation of sequences of interest by PCR) and Next-Generation-Sequencing (NGS) (sequencing and bioinformatics analysis of sequences of interest generated earlier by DNA walking). This allows to both detect and prove the presence of GMO in the food and feed chain by generating and characterizing sequences from their transgene flanking regions as well as unnatural element associations in their transgenic cassettes^1,8^. In a recent study by Fraiture *et al*.^1^, where this strategy was applied, the selected NGS platform was the PacBio RS II from Pacific Biosciences, which offers the opportunity to deal with heterogenic libraries and to provide long read-lengths (DNA fragments up to 60 kbp). Employing this NGS platform therefore allowed tackling some of the bioinformatics analysis issues encountered with the short read sequencing technologies, such as the Illumina platforms^8^.

Recently, a new NGS platform from Oxford Nanopore Technologies, the MinION, has become commercially available and has already been used for various bacterial, viral, yeast, animal, plant and human samples^9–16^. The MinION platform has been demonstrated to represent a promising alternative to the PacBio RS II platform due to its similar capacity to deal with heterogenic libraries and to provide long read-lengths (DNA fragments up to 200 kbp). Additionally, in contract to the PacBio RS II platform, the MinION platform also has the advantage of providing raw data in real-time and being more easily accessible by enforcement laboratories given its relatively affordable price and small size. Both these advantages are crucial when aiming to provide a fast answer in times of crisis as well as increasing the potential implementation of this strategy (combining qPCR screening, DNA walking, and MinION sequencing) in enforcement laboratories. However, the data analysis step still represents a major challenge, especially for someone without a bioinformatics background. In addition, the error rate observed with the Oxford Nanopore technology is slightly higher compared to the Pacific Biosciences technology. Moreover, tools for data analysis are less mature for the MinION data^17–21^.

To the best of our knowledge, the MinION platform was not yet reported to have been applied in the field of GMO detection. Therefore, the present paper is a proof-of-concept study aiming to investigate the potential and benefits of integrating the MinION platform into the strategy described above for the detection and characterization of GMO, including unauthorized ones. This advanced strategy was applied on an EU unauthorized genetically modified (GM) Bt rice, allowing to assess its feasibility, and to determine the possibility to prove the presence of GMO through the characterization of their transgene flanking regions and the unnatural associations of transgenic elements from their transgenic cassette, even when no *a priori* reference information is available.

## Results

**Figure 1:**
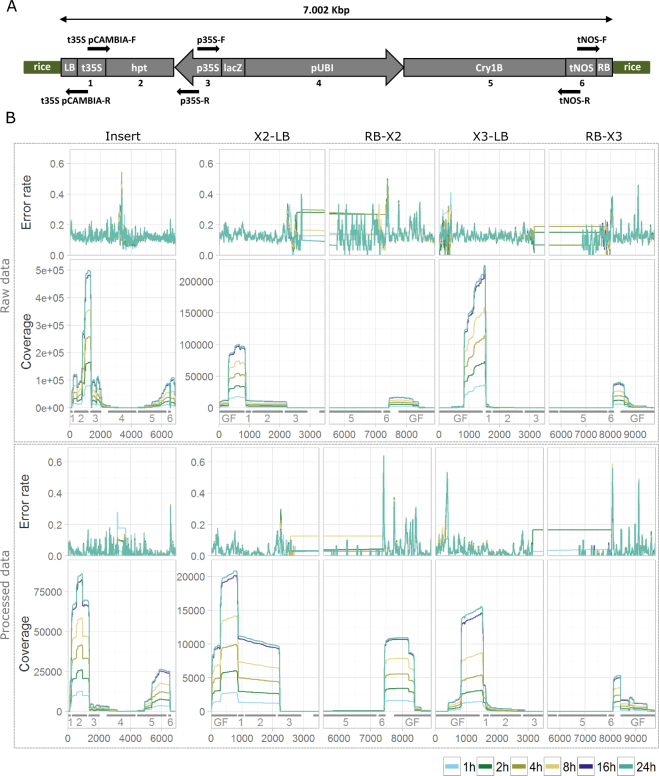
Coverage and error rate of raw and processed reads mapping to the transgene flanking regions and the transgenic cassette from Bt rice inserted on rice chromosomes II and III. (**A**) Schematic representation of the transgenic cassette of Bt rice. Each DNA walking method is illustrated by a black arrow. left border (LB); Cauliflower mosaic virus (CaMV) 35S terminator (t35S); hygromycin phosphotransferase gene (hpt); CaMV 35S promoter (p35S); LacZ alpha fragment (lacZ); maize ubiquitin promoter (pUBI); synthetic Cry1B gene (Cry1B); Agrobacterium tumefaciens nopaline synthase terminator (tNOS); right border (RB); rice genome (rice) [Schema adapted from 32]. (**B**) Coverage and error rate of raw reads and processed reads mapping on the common part of the insert, and the four unique transgene flanking regions from Bt rice. Read subsets obtained after 1, 2, 4, 8, 16 and 24 hours of sequencing are shown (see legend for color coding). Grey bars below the x-axis indicate the positions of the genomic flanks (GF) and of the functional genetic elements (1: t35S, 2: hpt, 3: p35S, 4: pUBI, 5: Cry1B, 6: tNOS). left border (LB); right border (RB); rice chromosome II (X2); rice chromosome III (X3).

Bt rice, used in this study, was previously characterized^22,23^. Bt rice presents two insertion sites of a transgenic cassette: one on the rice chromosome II and one on the rice chromosome III. The MinION sequencing run was performed on the PCR products generated from Bt rice using the bidirectional DNA walking methods anchored on the p35S, tNOS and t35S pCAMBIA transgenic elements (Fig. 1A).

### Technical aspects of the data analysis

**Figure 2:**
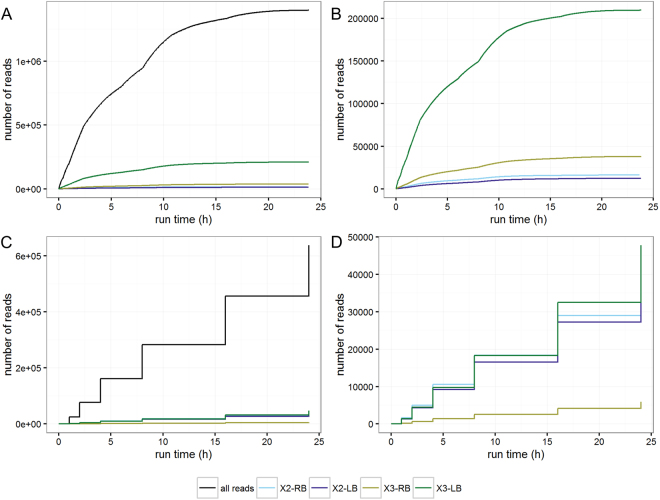
Acquisition profile of raw and processed reads generated by nanopore sequencing using the Oxford Nanopore MinION platform of a DNA library enriched via a DNA walking strategy applied to Bt rice. (**A**) Number of all raw reads, and raw reads mapping to the insertion sites on rice chromosomes II and III generated over time. (**B**) Focus on the number of raw reads mapping to the insertion sites on rice chromosomes II and III generated over time. (**C**) Number of all processed reads, and processed reads mapping to the insertion sites on rice chromosomes II and III generated over time. (**D**) Focus on the processed reads mapping to the insertion sites on rice chromosomes II and III generated over time. The ‘stepwise pattern’ of plots C and D compared to plots A and B is because for the former, only the total number of processed reads existing at 1, 2, 4, 8, 16 and 24 hours of sequencing were considered. All reads are represented by the black lines. The reads corresponding to the junctions between the rice chromosome II and the right border from the transgenic cassette (X2-RB) are indicated by the light-blue lines. The reads corresponding to the junctions between the rice chromosome II and the left border from the transgenic cassette (X2-LB) are indicated by the dark-blue lines. The reads corresponding to the junctions between the rice chromosome III and the right border from the transgenic cassette (X3-RB) are indicated by the yellow lines. The reads corresponding to the junctions between the rice chromosome III and the left border from the transgenic cassette (X3-LB) are indicated by the green lines.

The MinION sequencing run produced 1.6 million reads in 24 hours, corresponding to a total of 1.1 billion bases. Considering that the total size of the amplicons is expected to be less than 20 kbp (Fig. 1A, Additional file 2), this suggests adequate coverage for subsequent data analysis of all amplicons (e.g. >100×), despite the expected differences in their frequency in the sequenced library. The data acquisition was non-linear, with over 50% of the reads being available after only 8 hours (Table 1, Fig. 2A). To explore the possibility of using shorter sequencing times in subsequent experiments, thereby limiting either the sequencing costs by allowing to multiplex samples or the time needed to deliver results, we generated subsets of the sequencing data consisting of reads obtained after 1, 2, 4, 8, and 16 hours of sequencing. These data subsets were analyzed in parallel with the full dataset (24 hours) allowing to compare the results and to determine the optimal sequencing time (Table 1).

**Table 1:** Overview of raw and processed reads over sequencing time.

Raw data	1 h	2 h	4 h	8 h	16 h	24 h
Total reads	207417	425407	677698	961985	1356358	1422774
Mapped reads (%)	99.08	99.12	99.09	99.05	98.98	98.95
Error rate (%)	12.68	12.57	12.51	12.52	12.66	12.74
Minimal read size (bp)	100	100	100	100	100	100
Maximal read size (bp)	4458	4458	4458	4475	4475	4475
Average read size (bp)	511	509	508	505	501	499
**Processed data**	**1 h**	**2 h**	**4 h**	**8 h**	**16 h**	**24 h**
Total reads	24874	52139	84685	121185	173529	181544
Mapped reads (%)	99.13	99.09	99.04	99.02	99.00	99.00
Error rate (%)	2.92	2.83	2.84	2.83	2.84	2.85
Minimal read size (bp)	701	701	701	701	701	700
Maximal read size (bp)	2780	2790	2811	2817	2832	2852
Average read size (bp)	995	997	996	995	992	990

This following statistics were collected: read number, percentage of reads mapped to the reference sequences and related error rate, and minimum, maximum and average read size. Raw data: read data after removal of PCR adapters; Processed data: read data after removal of PCR adapters and retaining sequences of 100 bp or longer, error correction and quality trimming (see section “Materials and Methods”).

Mapping of raw reads to the sequences of the two transgenic cassettes inserted on the rice chromosome II and III demonstrated that the raw data had a highly stable mapping rate, error rate and read length profile over time, with a per base error rate of around 13% (Table 1). The mapping rate was at least 99% for all time points, indicating a relatively low level of non-specific amplification of the DNA walking procedure. To improve the error rates, raw reads were subjected to error correction and quality trimming, while opting to retain only reads exceeding 700 bp as all insertion sites were expected to be covered by a sufficient number of amplicons of at least this length (Fig. 1A). For processed reads, the maximal read length dropped from ~4.5 kbp to ~2.8 kbp for all data subsets, while the error rates improved from ~13% to 3% (Table 1, Fig. 1B). Mapping rates remained in the same range as for raw data.

As expected, the coverage was distributed unevenly over the reference, being highest near the t35S pCAMBIA, p35S and tNOS elements that contained the target-primer anchoring sites, from where the PCR amplification was initiated, but decreasing sharply to only a few reads in the middle of both inserts (Fig. 1B). This caused high fluctuations of the average error rate of the raw read data in the middle of the insert sequence (Fig. 1B), which was also mirrored to a certain extent in the processed data. Additional error rate peaks were observed with both raw and corrected data at homopolymer tracks and low complexity regions such as the ones located on chromosomes II and III near the junction with the right insert border (Fig. 1B).

### GMO detection feasibility and performance

To evaluate the feasibility of detecting GMO using the DNA walking strategy employing the MinION platform from Oxford Nanopore Technologies, we investigated two key aspects: the sequencing time required to reliably identify the transgene flanking regions as well as the possibility to characterize the different transgenic elements constituting the transgenic cassettes based on the generated sequences, without using any reference.

#### Detection of the transgene flanking regions

The detection of transgene flanking regions allows both the detection and identification of GMO present in the tested sample, which is crucial to prove the presence of GMO. Therefore, the minimum time required to obtain enough reads to reliably identify these sequences represents highly valuable information. As expected, Bt rice, as previously characterized^8,22–25^, contained two insertions sites for the transgenic cassette, one on rice chromosome II and one on rice chromosome III. Comparable to the data generation rate observed for the whole dataset, the number of sequences mapping to the junction regions increased rapidly until 8–10 hours (Fig. 2), after which the speed of sequence accumulation plateaued. Raw and processed read mapping to the sequences of the two transgenic cassettes inserted in rice chromosomes II and III indicated that only one hour of sequencing with the MinION platform was sufficient to identify all four insertion sites. Among the 207,417 raw reads obtained after 1 hour of sequencing, 2,633 reads mapped to the insertion site between rice chromosome II and the right border from the transgenic cassette while 1,505 reads mapped to the insertion site between rice chromosome II and the left border from the transgenic cassette. Similarly, 5,496 reads mapped to the insertion site between rice chromosome III and the right border from the transgenic cassette while 35,037 reads mapped to the insertion site between rice chromosome III and the left border from the transgenic cassette. The number of processed reads obtained after 1 hour of sequencing covering the four insertion sites was 1,636 reads for the insertion site between rice chromosome II and the right border from the transgenic cassette, 1,341 for the insertion site between rice chromosome II and the left border from the transgenic cassette, 190 for the insertion site between rice chromosome III and the right border from the transgenic cassette, and 1,426 reads for the insertion site between rice chromosome III and the left border from the transgenic cassette. The different number of raw reads covering the four insertion sites most likely results from differences in affinity of the primers used to amplify the corresponding DNA fragments, and the distance between the insertion sites and the annealing locations of the specific primers. The number of processed reads covering the sites additionally depends on the minimal length threshold chosen during the error correction and trimming steps. Although the number of processed reads covering the junction sites on chromosome III dropped more than expected, the resulting coverage was still adequate for the intended analysis, even for the 1 hour dataset. For the first sequencing hour, the percentage of correct bases in the consensus sequence of the junctions (defined as the 100 bp regions surrounding the insertion sites) was 99%, 97%, 94%, and 100% for the insertion site between rice chromosome II and the right or left border from the transgenic cassette and the insertion site between rice chromosome III and the right or left border from the transgenic cassette, respectively. This value did not differ noticeably with results obtained at longer sequencing times. The observed error rate for the consensus sequences of the junctions is sufficient to unambiguously identify Bt rice.

#### Characterization of the generated sequences without any reference sequence

In order to investigate the ability of the suggested DNA walking strategy to prove the presence of GMO without any *a priori* knowledge about the reference sequence, a data analysis workflow was tested to characterize the transgenic constructs and insertion sites via the annotation and partial assembly of the generated reads.

Given the results detailed above, all datasets starting from 1 hour of sequencing would be suitable for this purpose, but from a precautionary point of view, we have selected the data subset generated after 4 hours of sequencing. To reduce the dataset complexity prior to the annotation, the processed reads were first binned using a two-step clustering procedure. During the first round, clusters containing at least six reads that mutually aligned with 95% similarity along their entire length (≥99%) were retained, ensuring that any chimeric reads, potentially generated by the DNA library preparation and the Nanopore sequencing, were filtered^26,27^. The clustering returned 6,798 bins, consisting of 12.09 reads on average (with a maximum of 2,862 reads), out of which 1,600 clusters containing at least six reads were retained. During the second round, shorter reads that aligned with a minimal sequence similarity of 95% and a minimal alignment length of the shortest reads equaling 99% were binned with these longer reads thus further consolidating the data. Here, 200 clusters were formed with an average size of 7.14 reads (with a maximum of 168 reads), all of which were retained. The clustering procedure allowed decreasing the number of final reads more than 400-fold, limiting thus the computational power necessary to annotate the data.

**Figure 3:**
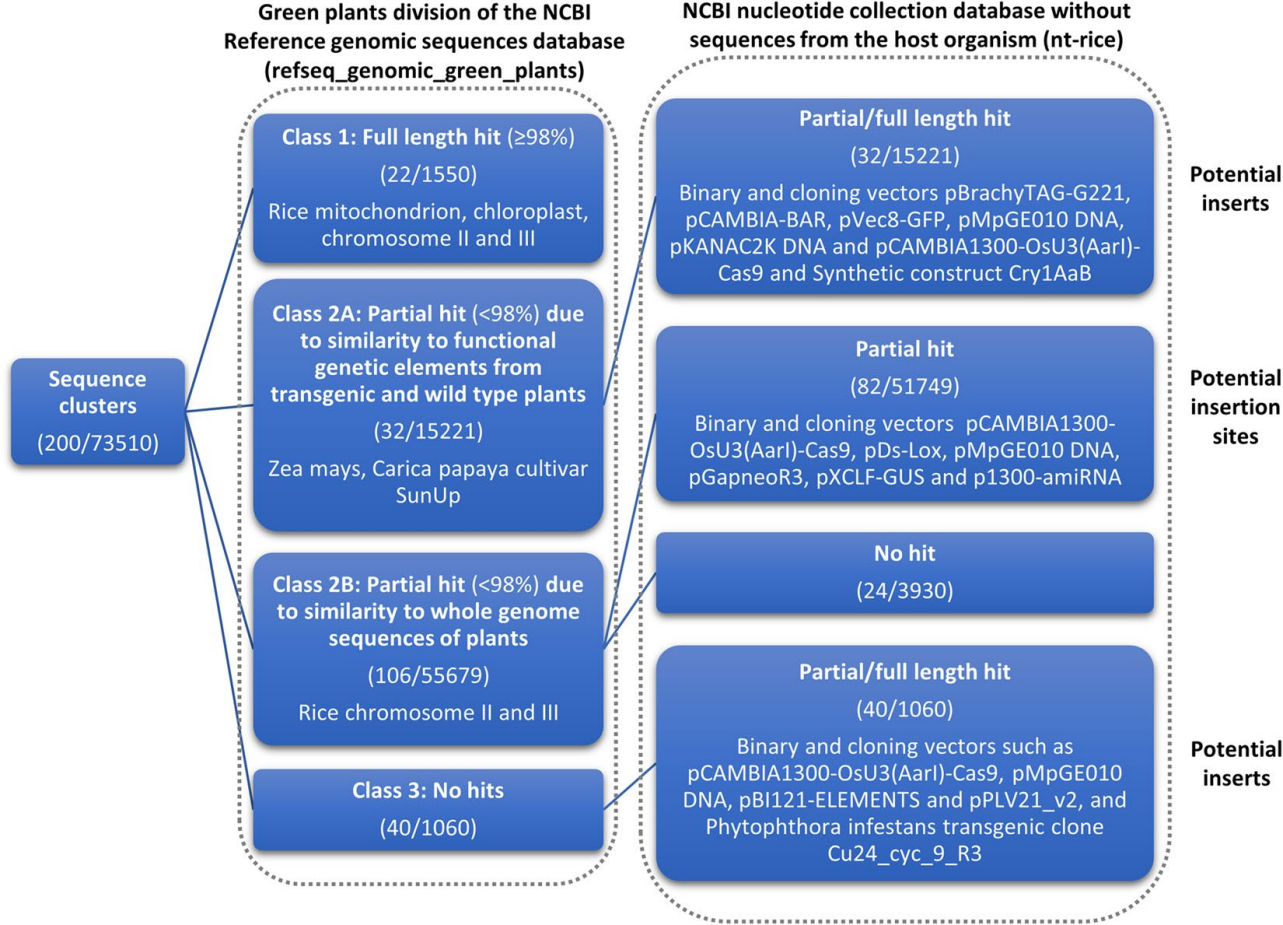
Workflow used for the annotation of the clusters. To annotate the clustered reads, the representative sequences of each cluster were blasted against both the green plants division of the NCBI Reference genomic sequences database (“refseq_genomic_green_plants”) and the NCBI Nucleotide collection database, excluding the Oryza sequences (“nt-rice”), using default blast parameters and a word size of 64. Initially, the sequences were subdivided as (class 1) sequences having a long hit (98% of the query length or more) against refseq_genomic_green_plants, (class 2) sequences having an intermediate-length hit (less than 98% of query length) against refseq_genomic_green_plants, further subdivided into (class 2A) sequences giving hits against the database due to similarity to functional elements such as promoters and coding regions from genomes of wild-type and transgenic plants and (class 2B) sequences having hits to the database due to hits against plant genomic sequences that did not encode functional genetic elements, and (class 3) sequences having no hits against refseq_genomic_green_plants. While class 1 was considered as non-informative and was not further processed, classes 2A, 2B and 3 were grouped according to their best hit against the nt-rice database: 2A and 3 sequences having full length or partial hits to the non-rice sequences were considered as potentially being obtained from internal parts of the transgenic insert, while sequences from the class 2B that aligned to the non-rice sequences were categorized as possibly containing the junction sequences of the transgenes. Sequences from class 2A having no hits against nt-rice could not provide any information. The numbers in brackets correspond respectively to the number of clusters with the total of processed reads associated.

The characterization of the clusters was automated according to the workflow schematized in Fig. 3, where representative sequences of each cluster were categorized according to the identity of their best hits against different NCBI databases. This workflow allows substantially reducing the hands-on time compared with manual blast analysis of each representative sequence of each cluster. More specifically, the sequence representative of every cluster was first aligned to the green plants division of the NCBI Reference genomic sequences database (“refseq_genomic_green_plants”) that contains curated genomic sequences. Based on the identity of the best hit reported for each representative sequence and the length of the hit, the clusters were grouped into three different categories (classes 1, 2 and 3), as indicated in Fig. 3. For classes 2 and 3, the representative sequences were subsequently aligned to the NCBI Nucleotide collection database from which the sequences belonging to the organism(s) identified in the first step, here the taxon *Oryza*, were excluded (“nt-rice”), allowing to further subdivide and characterise them. All blasts searches were carried out using a stringent minimal word size of 64 residues, limiting the non-specific hits but also resulting in more sequences that did not yield any hits against the two databases. Applying this workflow gave the following results.

Class 1 consisted of 20 clusters (1,287 reads) that had a nearly full length hit (≥98% of query length) against sequences from refseq_genomic_green_plants, more specifically to chloroplasts, mitochondria and chromosomes of *Oryza sativa*. These clusters were considered of no interest for further analysis as they were not informative and likely contained sequences from aspecific PCR amplification (Fig. 3; Additional file 3). Two additional clusters (263 reads) also showed hits against the *Oryza sativa* chloroplasts, but the aligned sequence was slightly shorter (~92%). They were added to this group after brief examination of the non-mapping overhanging ends in order to confirm that the overhangs did not consist of fragments of transgenic insert or vector.

Class 2 contained 138 clusters (70,900 reads) that had a partial length hit (<98% of query length) against the sequences from refseq_genomic_green_plants (Fig. 3; Additional file 3). This class was further divided into two sub-classes. For class 2A, 32 clusters (15,221 reads) were annotated as likely originating from the internal parts of a transgenic insert as they gave relatively short hits against various (non-rice) plant sequences when blasted against the refseq_genomic_green_plants database, and long hits with transformation and cloning vector sequences and a synthetic construct containing the *cry1AaB* gene (which is highly similar to the *cry1B* gene that is expressed in Bt rice) when blasted against the nt-rice database. Manual revision of the results indicated that for these sequences, hits obtained against the refseq_genomic_green_plants database were due to similarity of the transgenic elements located on the analyzed fragments, such as the promoter of the polyubiquitin gene from *Zea mays* or the terminator of nopaline synthase from *Agrobacterium tumefaciens*, to the corresponding elements in the genomes of the original donor plants (e.g. *Zea mays*) or transgenic plants (e.g. *Carica papaya* cultivar SunUp) (Fig. 3 - Class 2A; Additional file 3). For class 2B, 106 clusters (55,679 reads) produced a partial hit against rice chromosomes II or III. Among these 106 clusters, 82 clusters containing the majority of reads (51,749 reads) also produced hits against vector sequences when blasted against the nt-rice database, thus falling under the category of suspected transgene flanking region sequences. The remaining 24 clusters (3,930 reads) did not yield any hits against the nt-rice database and were not further analysed (Fig. 3 - class 2B; Additional file 3).

Class 3 encompassed 40 clusters (1,060 reads) that presented no hit against the refseq_genomic_green_plants database (Fig. 3; Additional file 3). When aligned to the nt-rice database, all the clusters showed a hit against various sequences from transformation and cloning vectors (i.e., pCAMBIA, pBI121-ELEMENTS and pDs-Lox). These clusters were also classified as potentially originating from the internal parts of the transgenic cassette (Fig. 3 - class 3; Additional file 3).

The obtained annotation facilitated a more detailed investigation and description of GMO, including the determination of the locations of the insertion sites by analysing the locations of the hits against rice genome, the reconstruction of the insert and the identification of the GM event. The locations of the hits of the presumable flanking regions (i.e., class 2B) against the rice genome showed presence of two clearly delineated insertion sites, respectively on chromosome II (right insert border at position 22,987,542 on chromosome II sequence NW_015379175.1 and left insert border at the position 22,987,632 on chromosome II sequence NW_015379175.1), and chromosome III (right insert border at position 23,615,166 on chromosome III sequence NW_015379176.1 and left insert border at position 23,615,178 on chromosome III sequence NW_015379176.1). These correspond to the two known insertion sites on rice chromosomes II and III previously described and verified by PCR^8,22–25^. The non-aligned overhangs of the reads were confirmed to match to the sequences of vectors, with the overwhelming majority aligning to T-DNA vector pDs-Lox (similar to right insert border) and cloning vector pCAMBIA1300-OsU3(AarI)-Cas9 (similar to left insert border) (Additional file 3).

Besides the two characterised insertion sites, a fraction of clusters partially aligned to an additional location on chromosome III (Additional file 4), with the overhanging parts of the clusters similarly corresponding to sequences of the transgenic cassette. The profile of the cluster alignment was, however, different from that observed in the previous cases: the sequences were clipped at several different locations relative to the chromosome without delineating a single insertion site. In addition, the sequences were fused to several different variations of transgenic insert sequences. This pattern was also observed for unclustered processed reads and for raw reads (Additional file 4), where an even bigger variety of fragments aligning to this chromosomal position showed that these sequences were likely chimeric products, potentially formed during the different rounds of PCR amplification and the Nanopore sequencing^26,27^. Most of the described sequences were discarded during the clustering procedure as they were strongly underrepresented in the dataset. Among the retained clusters, three combinations of sequences with a partial length hit to rice chromosome III and a partial length hit to the transgenic sequences and vector sequences represented the majority of the reads aligning at this position (Additional file 4). These three most prevalent combinations of sequences were tested by PCR and could be proven to be absent from the Bt rice genome (absence of amplification; data not shown).

**Figure 4:**
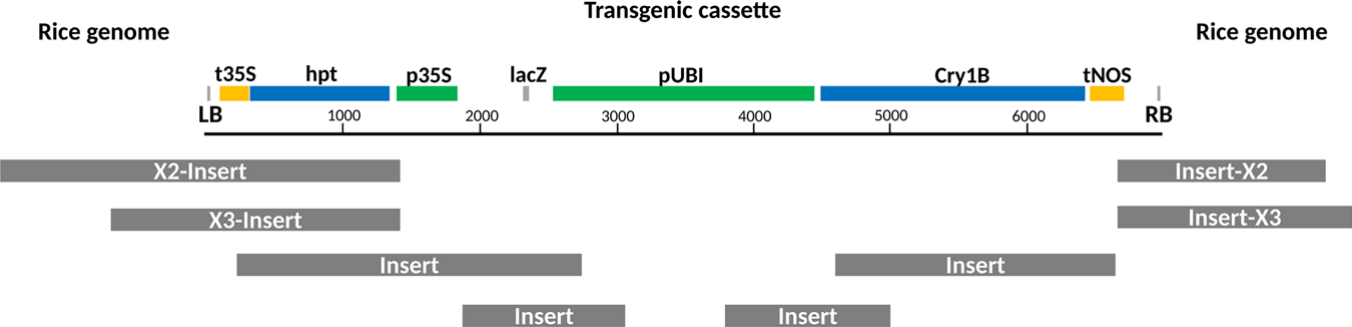
Illustration of the assembled flanking regions and longest representative insert fragments. The grey bars below the transgenic cassette represent the longest representative fragments obtained for the insert, the transgene flanking regions on the rice chromosome II (X2-insert and insert-X2) and the transgene flanking regions on the rice chromosome III (X3-insert and insert-X3). left border (LB); Cauliflower mosaic virus (CaMV) 35S terminator (t35S); hygromycin phosphotransferase gene (hpt); CaMV 35S promoter (p35S); LacZ alpha fragment (lacZ); maize ubiquitin promoter (pUBI); synthetic Cry1B gene (Cry1B); Agrobacterium tumefaciens nopaline synthase terminator (tNOS); right border (RB).

Further, the longest and/or the largest clusters were selected for each of the confirmed junction sequences and reassembled to obtain the longest possible flanking region (Fig. 4). The sequences from classes 2A and 3 that were considered to originate from the transgenic inserts were subdivided into categories according to the identity of their best hit against the nt-rice database. Reads within the different subgroups were then realigned to each other and to the junction regions using blast to remove all sequences that aligned with other sequences along their entire length and determine the relative positions of the retained fragments. This allowed to recover a considerable fraction of the insert and junction sequences (Fig. 4).

Finally, with the aim of defining the EU authorization status of the identified GMO, a region of 100 residues around each junction was excised and aligned to (1) the JRC-Amplicons database^28^ and (2) the patent (pat) database of NCBI. The former database contains transgenic junction sequences from all authorized GM plants in Europe, and the latter contains transgenic junction sequences of all patented GM plants. None of the excised junctions originated from authorized or patented GM plants, as none of the junctions gave a full-length alignment with any of the sequences present in the databases. As a control, the same sequences were aligned to the NCBI Nucleotide collection database, which is known to contain two transgene flanking regions out of four: one between the right border from the transgenic cassette and rice chromosome II [GenBank: KT184679] and one between the right border from the transgenic cassette and rice chromosome III [GenBank: KT184678]^23^. As expected, both right-border junctions retrieved correct hits with either KT184679 or KT184678 spanning over 90% of the query length. The two left borders that were not represented in the database on the other hand did not result in any hits longer than 50%.

## Discussion

The detection of unauthorized GMO represents a crucial challenge in terms of worldwide public health due to the lack of traceability in the food and feed chain and information regarding potential health risks. More precisely, in many countries around the world, the GMO approval and the compliance declaration with the related regulations are depending on a mandatory risk assessment, which is not carried out for unauthorized GMO. In order to strengthen the current GMO detection system, essentially consisting of qPCR analysis that targets GM elements from transgenic cassettes as well as EU authorized event transgene flanking regions, the added value of NGS technologies has already been investigated^1,29,30^. Amongst potential targeted NGS approaches, an enrichment of the DNA library using DNA walking anchored on key GM elements has recently been proposed. For this *a priori* approach, the selection of the key GM elements is crucial. For instance, in targeting both p35S and tNOS, most of EU authorized GMO (~70%) and approximately 90% of EU unauthorized GMO are covered. If necessary, additional DNA walking methods anchoring on other GM elements, previously positive in qPCR screening analysis, could also be developed in order to obtain new information. However, the present approach is clearly not applicable for GMO which do not possess any of the GM elements targeted during the qPCR screening analysis. For this purpose, a non *a priori* approach, such as whole genome sequencing (WGS), is more suitable^1,8,29,31^.

Regarding the sequencing platforms, we are moving away from technologies requiring DNA libraries composed of short sequences of the same size, such as Illumina, to reduce the potential complexity in the bioinformatics analysis of samples containing several different GMO^1,8^. Currently, two NGS technologies, namely single-molecule real-time sequencing marketed by Pacific Biosciences and nanopore sequencing marketed by Oxford Nanopore, offer the possibility to sequence long fragments of different sizes, allowing to sequence the amplicons generated by DNA walking (i.e., size range from 200 bp to 4 kbp) in its entirety. Although the Pacific Biosciences technology has recently been successfully tested in the GMO detection field, no study using Oxford Nanopore technology for this purpose has, to the best of our knowledge, already been published^1,8,26,31^. Given the potential benefits of the Oxford Nanopore technology (i.e., raw data delivered in real-time, a small and portable device, relatively easy to manipulate, and attractively priced), we have investigated the feasibility of using the MinION platform to evidence the presence of GMO, including unauthorized ones. Based on the results from the present study, our main findings are described below.

First, at a technical level, the data analysis steps required to collect and analyze the raw data were in general not user-friendly for non-bioinformaticians and could be challenging even for bioinformaticians. During our period of data analysis (middle of 2017), the Oxford Nanopore supporting information was often incomplete and some of the recommended tools were updated or replaced regularly. For instance, real-time basecalling with Metrichore was not longer possible after our basecalling analysis. The implementation of the DNA walking strategy coupled with the MinION platform will therefore require the development of adapted pipelines and reliable data processing tools that allow fast, stable, intuitive and relatively automated data analysis, independent from tools made available by Oxford Nanopore. The computational burden of bioinformatics analysis is currently also high and may currently not be available for all enforcement laboratories. An additional inconvenience is that MinION data suffer from relatively high error rates (i.e., here around 13%) which could interfere for instance with efficient clustering of the raw data and its annotation based on database matches. Therefore, we have performed error correction of the raw reads with Canu prior to clustering and annotation, thus simplifying the downstream data analysis steps. The processed reads demonstrated substantially higher accuracy (~3%), but still contained a number of errors compared to the known reference, especially at the locations containing homopolymer tracks and low complexity regions. Even if Oxford Nanopore announced a short time ago the release of a new version (v2) of the Albacore basecalling software, which is expected to produce more accurate results at these repetitive regions, possibly alleviating this particular problem in the future, this new version (v2) was not available at the time of our data analysis. In addition to our bioinformatics analysis, other strategies can alternatively be investigated to optimize the data analysis for either running time or accuracy, involving for instance multiple rounds of error correction or the use of alternative error correction tools. However, the bioinformatics analysis used in this study allowed a sufficient accuracy for detailed characterization of the data since, in the context of GMO detection, large modifications in the genome are expected conversely to, for instance, SNP identification. Therefore, even if sequences of interest contained errors (around 3%), they will still allow proving the presence of GMO.

Second, at the level of feasibility of the proposed approach, the MinION platform and the associated bioinformatics analysis, applied to DNA fragments enriched by DNA walking anchored on key targets, showed to be clearly suitable for the characterization of GMO, including unauthorized ones. Indeed, the expected four transgene flanking regions as well as large parts from the transgenic cassette were successfully retrieved, allowing to unambiguously demonstrate the presence of GM material in the sample. However, it is at present still advised to verify any newly discovered transgene flanking regions by, for instance, PCR assays. Concerning the sequencing time required to observe the four expected transgene flanking regions, the dataset generated after only 1 hour of sequencing proved to be already adequate for data analysis, although there were substantial differences in coverage at this time point for the four different transgene regions (Fig. 2). Longer sequencing times led to relatively small improvements in terms of error rate and sequence length compared to the first sequencing hour. Therefore, from a precautionary point of view, the four hour dataset was selected for the final analysis, in order to have an ideal balance between a higher coverage, increasing with the sequencing time, and a fast delivery of the results. These observations suggest that for less complex samples (i.e., such as here with a sample composed exclusively of one GM event), short sequencing times such as 1 hour to 4 hours can be applied safely, or a larger number of samples can be analyzed in a single run, thus reducing data acquisition time and cost of analysis. For samples containing a low amount of GMO as well as more complex GMO mixtures, additional assays with the MinION device have to be performed to define the shortest advisable sequencing time. However, given the present promising results, the analysis of more complex samples is expected to be potentially feasible.

Third, at the level of interpretation, the characterization of the DNA fragments could become cumbersome without any reference. More precisely, the library preparation produced by DNA walking with multiple primers can become relatively complex, containing multiple amplicons, some of which can have large overlapping regions. In addition, the library complexity is expected to increase rapidly in case the original sample consists of a mixture of different GM events with similar transgenic elements or one GM event with multiple insertion sites, such as in the present analysis as well as in stacked events. Because of this complexity, manual characterization of the processed data is very tedious and therefore rapidly becomes a limiting step. In this study, we have thus applied a reference free stepwise semi-automated annotation workflow allowing to characterize the representative amplicons based on clustering of reads. In its current implementation, the workflow permitted to categorize the sequences, detect the presence of transgenic vector fragments and identify fragments corresponding to flanking regions (Fig. 3, Additional file 3), and to determine the EU authorization status of the identified GMO through comparison with publicly available databases from NCBI and JRC Amplicons. In the future we believe that a nearly complete automation of the data annotation workflow will be possible. In any case, the current analysis has demonstrated several bottlenecks where the annotation workflow would substantially benefit from the availability of a specialized database, containing sequences of commonly used transgenic elements, inserts and transgenic vectors from at least EU authorized GM events. For instance, it will allow to automate the manual revision of the sequences from the class 2 to identify which sequences contain the junction region, and which merely give a hit to the refseq_genomic_green_plants database because of similarity with functional genomic elements from the plants that are not present in the tested sample. Besides, it will allow a more detailed characterization of the detected sequences originating from transgenic vectors. Moreover, even if the junction between a transgenic insert and the host genome will not have been recovered, the observation of sequences belonging only to the transgenic cassettes (unnatural associations of transgenic elements) will even so indicate the presence of GMO. In addition, the detection of different combinations of transgenic elements compared to the known combinations in transgenic cassettes from EU authorized GMO will also indicate the presence of unauthorized GMO. In case previously unknown GM events are characterized, the sequences from their transgene flanking regions could also be used in order to develop novel qPCR GM-event specific methods that could be used in GMO routine analysis by the enforcement laboratories.

## Materials and Methods

### Plant material

Rice grains from transgenic Bt rice (*Oryza sativa L. Japonica cv Ariete*), transformed by *Agrobacterium tumefaciens* with the binary vector pCAMBIA 1300 containing the synthetic Cry1B gene from *Bacillus thuringiensis* to confer an insect resistance, was used in this study (Fig. 1A)^32^. Based on its previous characterization, Bt rice has two insertions sites, one on the rice chromosome II and one on the rice chromosome III, where the transgenic cassette has been integrated (Fig. 1A)^8,22–25^. The used reference sequences of the transgenic cassette and the four transgene flanking regions were previously generated based on a WGS assay applied on the Bt rice [see point “Generation of plots and descriptive statistics of the data”; 25].

### DNA extraction, concentration and purity

Rice grains were manually ground to a fine homogenous powder and used for DNA extraction using a C-hexadecyl-Trimethyl-Ammonium-Bromide (CTAB) based procedure (ISO 21571) in combination with the Genomic-tip20/G (QIAGEN, Hilden, Germany) procedure adapted from the EU-RL GMFF validated method^33,34^. This DNA extraction method comprises four successive steps: extraction of proteins, polysaccharides and organic components; precipitation of DNA in the presence of CTAB; purification of DNA using a tip20 column; and precipitation of DNA with isopropanol^33,34^. DNA concentration was measured by spectrophotometry using the Nanodrop^®^ 2000 (ThermoFisher, DE, USA) and DNA purity was evaluated using the A260/A280 and A260/A230 ratios.

### DNA walking

The DNA walking approach, allowing to isolate the unknown sequences flanking to the known sequences, was carried out as previously described^2–24^ (Additional file 1). First, a target-specific primer (a) and one kind of the degenerated random tagging primer (DRT) mixes (A–D) were applied to amplify the sequences of interest. In a second and third semi-nested PCR, target-specific primers (b and c) were combined, respectively, with universal tagging primers (UAP-N1 and UAP-N2) in order to increase the yield of the sequences of interest and to decrease the background. Using the p35S-F, p35S-R, tNOS-F, tNOS-R, t35S pCAMBIA-F and t35S pCAMBIA-R DNA walking methods, 100 ng of DNA from a 100% Bt rice sample, corresponding to 200 000 haploid genome equivalents (HGE) of Bt rice, was analyzed.

### Library preparation and sequencing

All PCR products generated by the DNA walking methods were pooled and then purified using the AGENCOURT® AMPURE® XP Kit (Beckman Coulter Life Sciences, Indianapolis, USA), according to the manufacturer’s instructions. The profile of the sample was analyzed using the Agilent High Sensitivity D5000 kit through the Agilent 4200 TapeStation system (Agilent, Waldbronn, German), according to the manufacturer’s instructions (Additional file 2). The concentration of the sample was measured using the Qubit dsDNA HS Assay Kits and a Qubit 3.0 Fluorometer (Life Technologies, Belgium), according to the manufacturer’s instructions. The library was prepared from 1 µg of the sample composed of the pooled PCR products, generated by the DNA walking methods, using the 1D amplicon by ligation sequencing kit (SQK-LSK108; Oxford Nanopore Technologies, Oxford, UK), according to the manufacturer’s instructions. Briefly, the double-stranded DNA fragments were initially end-repaired and dA-tailed before being ligated to adapters. The prepared library was subsequently loaded into the MinION flow cell (106 R9) of the MinION sequencing device (Oxford Nanopore Technologies, Oxford, UK). The sequencing run was performed during 24 hours.

### Sequencing data collection and generation of read subsets

The basecalling of the raw generated data, in fast5 format, was performed via the cloud-based Metrichor™ Agent using the 1D basecalling workflow. The individual fastq files were extracted from the base-called fast5 files with poretools (version 0.6.0)^35^. In addition, the sequencing start times of individual reads were collected from the fast5 files using a custom python script based on the h5py library (version 2.6.0)^36^. The obtained information was used to extract read subsets generated upon 1, 2, 4, 8 and 16 hours from the total sequencing data. All subsequent sequence data analysis steps described in 4.6 were applied in parallel on the generated sequencing data subsets and on the total dataset.

### Sequencing data analysis

MinION adapters were removed with Porechop (version 0.2.2) using default parameters except for a minimal trim size of 4 bp and the middle_threshold lowered to 0.75, which is advised in the program’s manual to improve recognition of adapters inside the reads^37^. Next, DNA walking adapters (GGAAGCAGTGGTATCAACGCAGAGTGGCCATTACGGCCGAACACGCGTCGTTTACCT) were removed using Cutadapt (version 1.14) with an error rate of 0.30, a minimal match length of 5 bp, allowing the removal of multiple adapter occurrences within a single read and only retaining reads that are at least 100 bp long^38^. The adapter-trimmed reads were subjected to error correction using Canu (version 1.5) using the following parameters: a minimal read length of 700 bp, a minimal overlap length of 700 bp, an estimated genome size of 100 kb, a corOutCoverage of 20000 and raw-nanopore reads as the input read type^39^. The read correction algorithm of Canu corrects the longer reads present in the dataset using the shorter ones. The genome size estimate is used to calculate the fraction of the longest reads to correct, with the fraction of the longest reads chosen so that they cover the genome up to the value given by corOutCoverage. The selection of a high corOutCoverage and a larger than expected approximate genome size ensures that all reads in the dataset are corrected, as suggested in the Canu manual for cases where the library contains DNA molecules with a substantially different copy number and size, such as plasmids or amplicons. Error-corrected reads were trimmed with the trim reads function of Canu, with a minimal read length of 700 bp, a minimal overlap length of 700 bp, an estimated genome size of 100 kb and a corOutCoverage of 20000. Trimming allows to remove the low support bases that were retained during the error correction. An additional round of adapter trimming with cutadapt was performed to remove remaining DNA walking adapter sequences. The datasets were then clustered in a two-step clustering procedure using CD-HIT (version 8.22)^40,41^. During the first clustering step, a global alignment was carried out with a minimal sequence similarity of 95%, a minimal length similarity of 99%. Only clusters containing at least six reads were retained. This way, the underrepresentared reads were discarded as well as any potential chimeric reads, potentially generated by the DNA library preparation and the Nanopore sequencing, were filtered. The output of the first clustering step was subjected to a second clustering round applying a local alignment with a minimal sequence similarity of 95% and a minimal alignment length of the shortest read equaling 99% of its length.

### Generation of plots and descriptive statistics of the data

To evaluate the number of reads containing one of the four transgene flanking regions, reads were mapped on the reference sequences consisting of the transgenic inserts and several hundreds of base-pairs of the corresponding flanking regions using BWA-MEM (version 0.7.15) using the option -ont2d for MinION reads^42^. The reads covering 100 bp around the transgene flanking regions were extracted using samtools (version 1.3.1). The consensus sequence of the region, and the corresponding number of correct bases were calculated using the consensus.py script^43^ that was applied on processed reads with a minimal coverage threshold of 2 reads. To calculate the sequencing coverage and error rate of the available reference, consisting of the two transgenic inserts with the corresponding flanks, reads were mapped using BWA MEM and the statistics were extracted using Pysamstats (version 1.0.0, with pysam version 0.11)^44^. General summary statistics were obtained using Qualimap 2 (version 2.2.1)^45^.

### Annotation of the clustered reads

To annotate the clustered reads, the representative sequences of each cluster were blasted against both the green plants division of the NCBI refseq_genomic database^46^ and the NCBI nt database^47^, excluding the *Oryza* sequences, using default blast parameters and a word size of 64. The results were parsed using the SearchIO function of BioPython (version 1.65)^48^ and grouped based on the identity of the best hits against the two databases (Fig. 3). Initially, the sequences were subdivided as (class 1) sequences having a long hit (98% of the query length or more) with the genomic sequences of green plants, (class 2) sequences having an intermediate-length hit (less than 98% of query length) with the genomic sequences of green plants, further subdivided into (class 2A) sequences giving hits against the database due to similarity to functional elements such as promoters and coding regions from genomes of wild-type and transgenic plants and (class 2B) sequences having hits to the database due to hits against plant genomic sequences that did not encode functional genetic elements, and (class 3) sequences having no hits against the genomic sequences of green plants. While class 1 was considered as non-informative and was not further processed, classes 2A, 2B and 3 were grouped according to their best hit against the nt database excluding *Oryza* subdivision: 2A and 3 sequences having full length or partial hits to the non-rice sequences were considered as potentially being obtained from internal parts of the transgenic insert, while sequences from the class 2B that aligned to the non-rice sequences were categorized as possibly containing the junction sequences of the transgenes. Sequences from class 2A having no hits, could not provide any information.

To finally describe the amplicons that were present in the DNA walking library, the positions of the potential flanking sequences on the rice chromosomes were examined, revealing the insertion sites. Longest and/or most populated amplicons were selected for each junction, and reassembled using minimus2 tool from AMOS package (version 3.1.0) with default parameters^49^. The potential insert sequences were further arranged according to their hits against the insert parts of the obtained junction sequences, to each other, or based on the hits that these sequences produced against the nt database excluding *Oryza*.

Further, sequences corresponding to the insertion sites were excised by taking the region of 100 bp around the midpoint between the hits against the vector and the rice chromosome. The obtained fragments were aligned to the JRC-Amplicons, NCBI pat and the NCBI nt databases. The hits were reviewed to verify the possibility to identify an unknown GMO event using the proposed strategy.

### Verification of transgene flanking regions by PCR amplification

All the presumable transgene flanking regions were verified by PCR. The four expected transgene flanking regions were previously confirmed by PCR^22,23^. Here, the three most prevalent unexpected transgene flanking regions observed through the bioinformatics analysis were tested. These unexpected transgene flanking regions presented a partial length hit to rice chromosome III while also containing a part that showed similarity to the transgenic cassette. Using the software Primer3, one primer (AACATACCAGCAGGGTCCAG) was designed to anneal to rice chromosome III sequence near the targeted transgene flanking regions. This primer was individually combined with the p35S-R c, t35S-R c and tNOS-F c primers, whose binding sites were present of the tested sequences. A standard 25 µl reaction volume was applied containing 1 U of Taq DNA Polymerase recombinant (Invitrogen, Gent, Belgium), 1X PCR Buffer (Invitrogen, Gent, Belgium), 1.5 mM of MgCl_2_ (Invitrogen, Gent, Belgium), 0.2 mM of dNTPs (Invitrogen, Gent, Belgium), 250 nM of each primer (Eurogentec, Liège, Belgium) and 5 µl of DNA (5 ng/µl). The PCR program consisted of a single cycle of 10 min at 94 °C (initial denaturation) followed by 35 amplification cycles of 45 sec at 94 °C (denaturation), 30 sec at 55° or 60 °C (annealing) and 90 sec at 72 °C (extension) and finishing by a single cycle of 10 min at 72 °C (final extension). The run was performed on a Swift MaxPro Thermal Cycler (Esco, Analis, Rhisnes, Belgium). The PCR product was analyzed by electrophoresis on a 1% agarose gel (INVITROGEN, CA, USA) (100 V, 400 mA, 60 min).

### Data availability

All data generated or analyzed during this study are included in this published article [and its supplementary information files] or is available from the corresponding author.

## Electronic supplementary material

Supplementary data
