# Supplementary material for: Nanopore sequencing technology: a new route for the fast detection of unauthorized GMO
Source: Sci Rep. 2018 May 21;8:7903. doi: 10.1038/s41598-018-26259-x (PMC5962636; doi:10.1038/s41598-018-26259-x)
Supplement: Supplementary file 1 — Supplementary data [file 41598_2018_26259_MOESM1_ESM.docx]

**Nanopore sequencing technology: a new route for the fast detection of unauthorized GMO**

**Marie-Alice Fraiture, Assia Saltykova, Stefan Hoffman, Raf Winand, Dieter Deforce, Kevin Vanneste, Sigrid CJ De Keersmaecker, Nancy HC Roosens**

Additional file 1: Oligonucleotide sequences used for qPCR and DNA walking assays.

| **Methods** | **Oligonucleotide names** | **Oligonucleotide sequences** | **References** |
| --- | --- | --- | --- |
| **DNA Walking** | p35S-F a | GGGTCTTGCGAAGGATAGTG | 5 |
|  | p35S-F b | TGTGCGTCATCCCTTACGTCAGT | 23 |
|  | p35S-F c | TATCACATCAATCCACTTGCTTT | 5 |
| **DNA Walking** | p35S-R a | AAAGCAAGTGGATTGATGTGATA | 5 |
|  | p35S-R b | ACTGACGTAAGGGATGACGCACA | 23 |
|  | p35S-R c | CACTATCCTTCGCAAGACCC | 5 |
| **DNA Walking** | tNOS-F a | GATTAGAGTCCCGCAATTATACATTTAA | 5 |
|  | tNOS-F b | TTAATACGCGATAGAAAACAAAAT | 23 |
|  | tNOS-F c | AAATATAGCGCGCAAMCTAGGATAA | 5 |
| **DNA Walking** | tNOS-R a | TTATCCTAGKTTGCGCGCTATATTT | 5 |
|  | tNOS-R b | ATTTTGTTTTCTATCGCGTATTAA | 23 |
|  | tNOS-R c | TTAAATGTATAATTGCGGGACTCTAATC | 5 |
| **DNA Walking** | t35S pCAMBIA-F a | CGGGGGATCTGGATTTTAGTA | This study, adapted from 22 |
|  | t35S pCAMBIA-F b | GGGTTTCTTATATGCTCAACAC | This study, adapted from 22 |
|  | t35S pCAMBIA-F c | GAGCGAAACCCTATAGGAACCCT | This study, adapted from 22 |
| **DNA Walking** | t35S pCAMBIA-R a | TACTAAAATCCAGATCCCCCG | 22 |
|  | t35S pCAMBIA-R b | GTGTTGAGCATATAAGAAACCC | 22 |
|  | t35S pCAMBIA-R c | AGGGTTCCTATAGGGTTTCGCTC | 22 |

| **Additional file 2:** Profile, created using the Agilent 4200 TapeStation system, of the pooled PCR products generated from the p35S-F, p35S-R, tNOS-F, tNOS-R, t35S pCAMBIA-F and t35S pCAMBIA-R DNA walking methods applied on the 100% Bt rice sample. The y and x axis indicate respectively the sample intensity in FU (fluorescence unit) and the amplicon sizes in bp. The Agilent High Sensitivity D5000 assay includes a lower and upper marker. |
| --- |
| 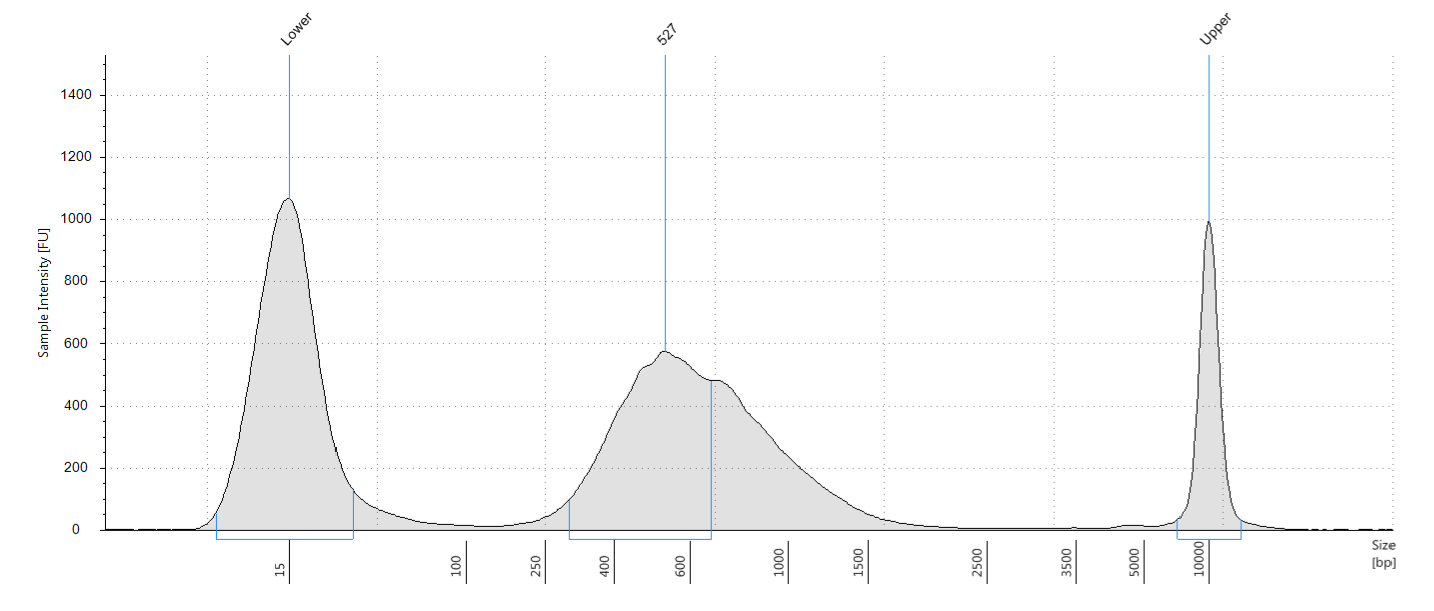 |

|  |
| --- |
| **Additional file 3:** Annotation workflow of the processed and clustered sequences. L and %L respectively indicate the average length of the alignment in base pairs and average percentage of the query sequence covered by the alignment. Standard deviation is indicated near the average values. |

| Raw data aligned to the rice chromosome II insertion site |
| --- |
| 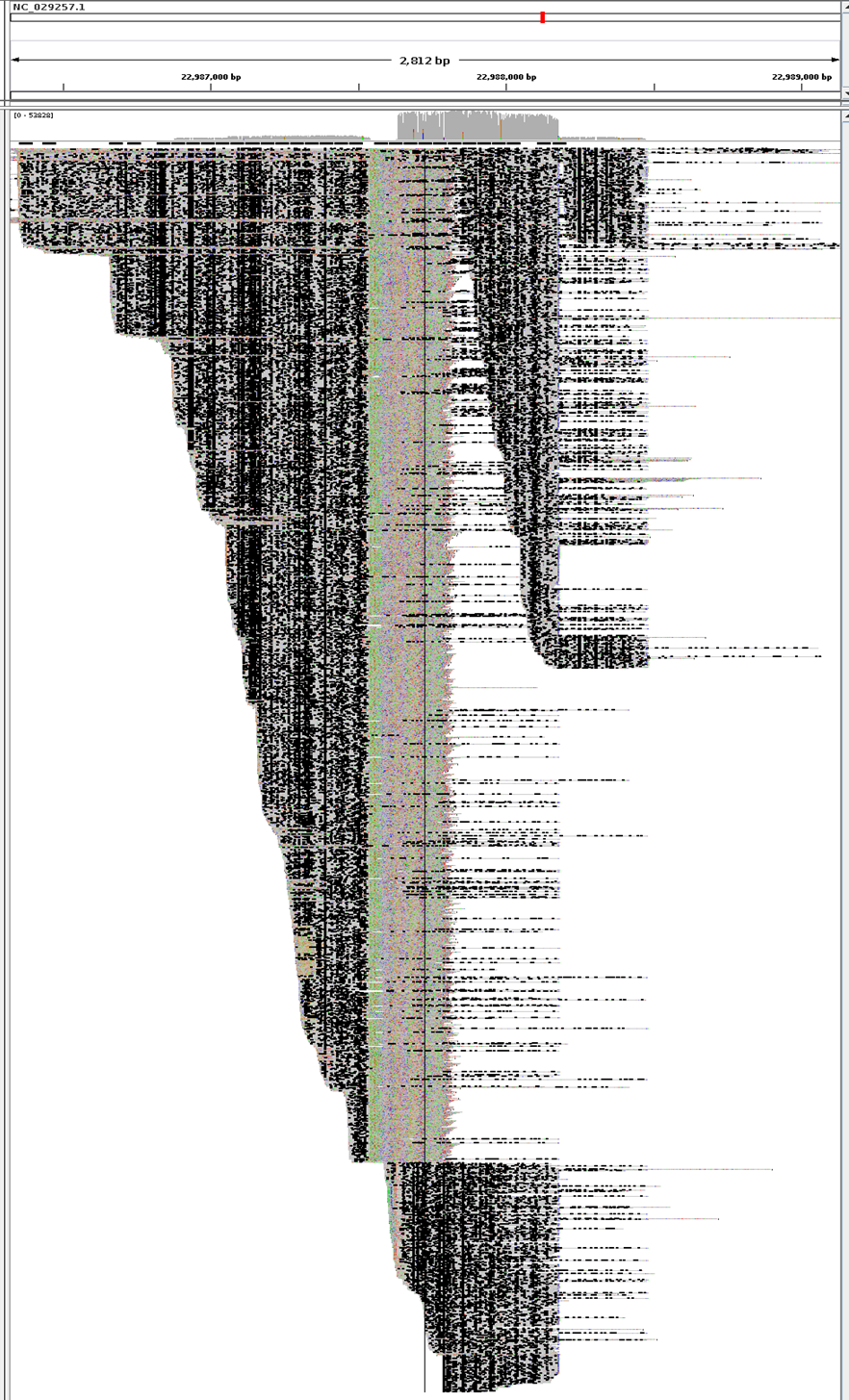 |
| Processed data aligned to the rice chromosome II insertion site |
| 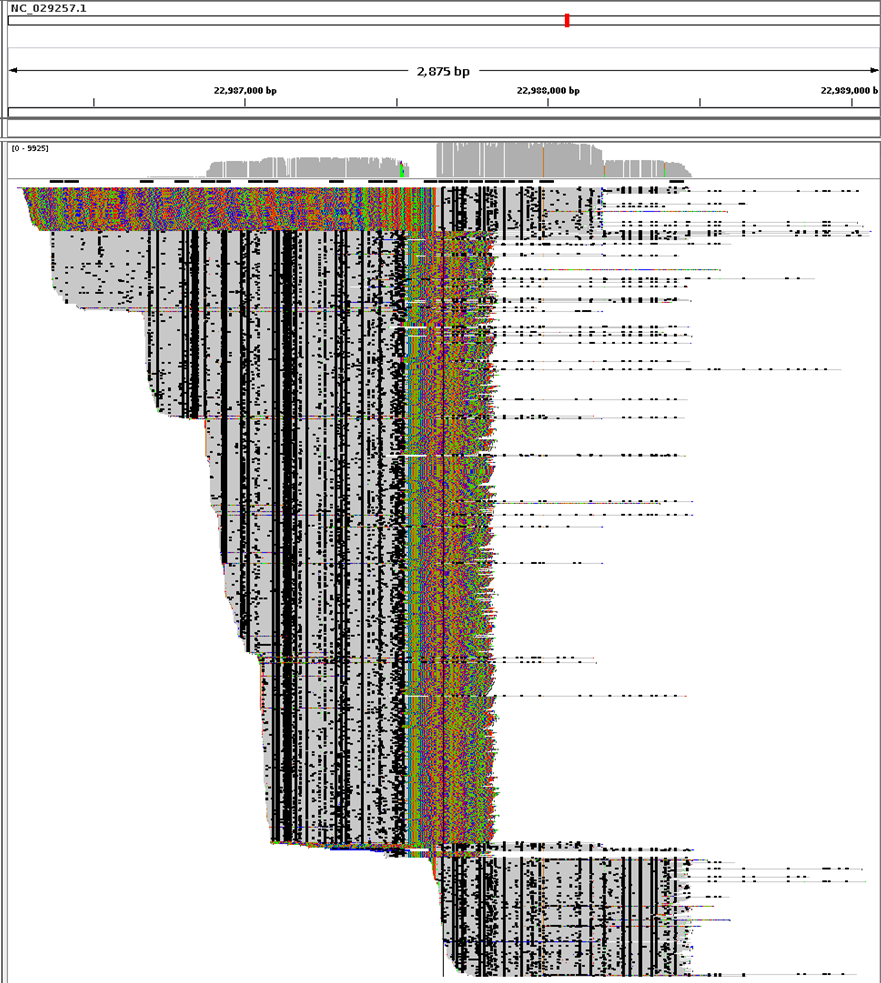 |
| Clustered data aligned to the rice chromosome II insertion site |
| 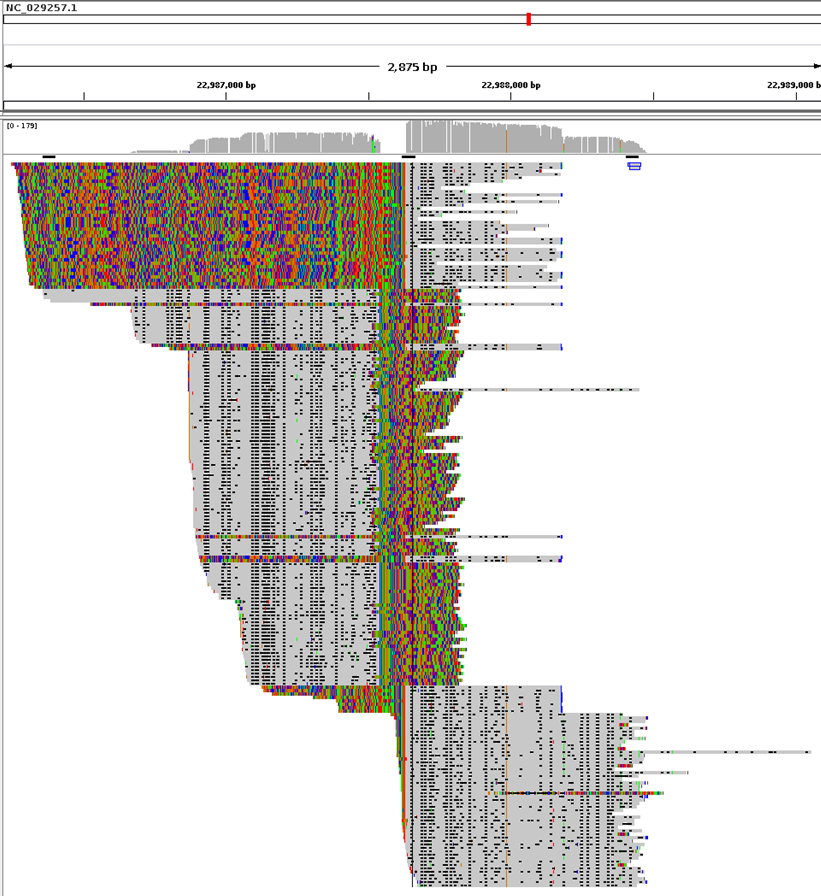 |
| Raw data aligned to the rice chromosome III insertion site |
| 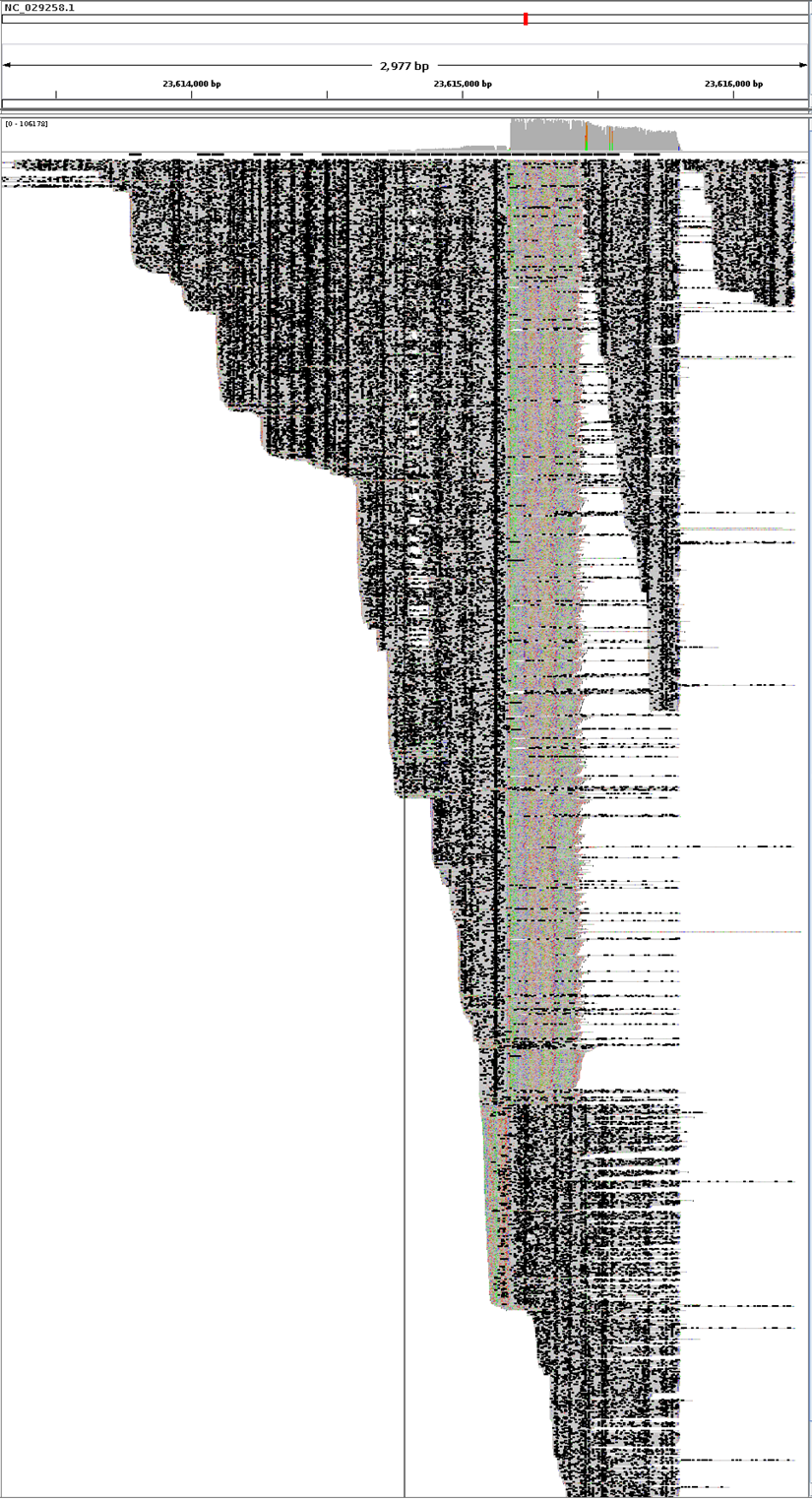 |
| Processed data aligned to the rice chromosome III insertion site |
| 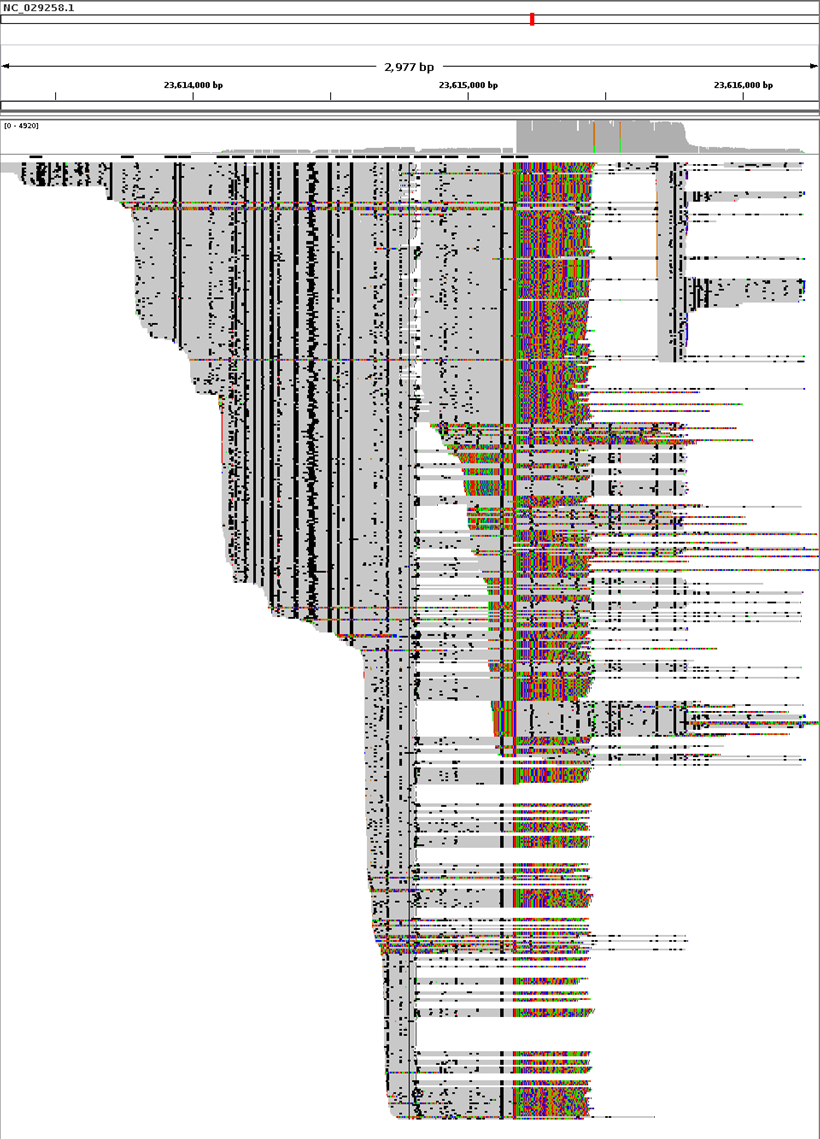 |
| Clustered data aligned to the rice chromosome III insertion site |
| 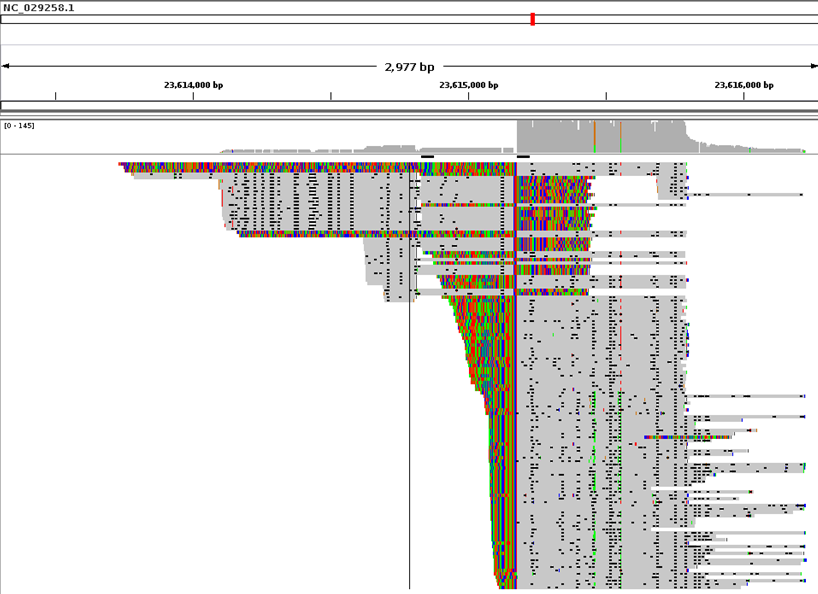 |
| Raw data aligned to the rice chromosome III (aspecific amplification) |
| 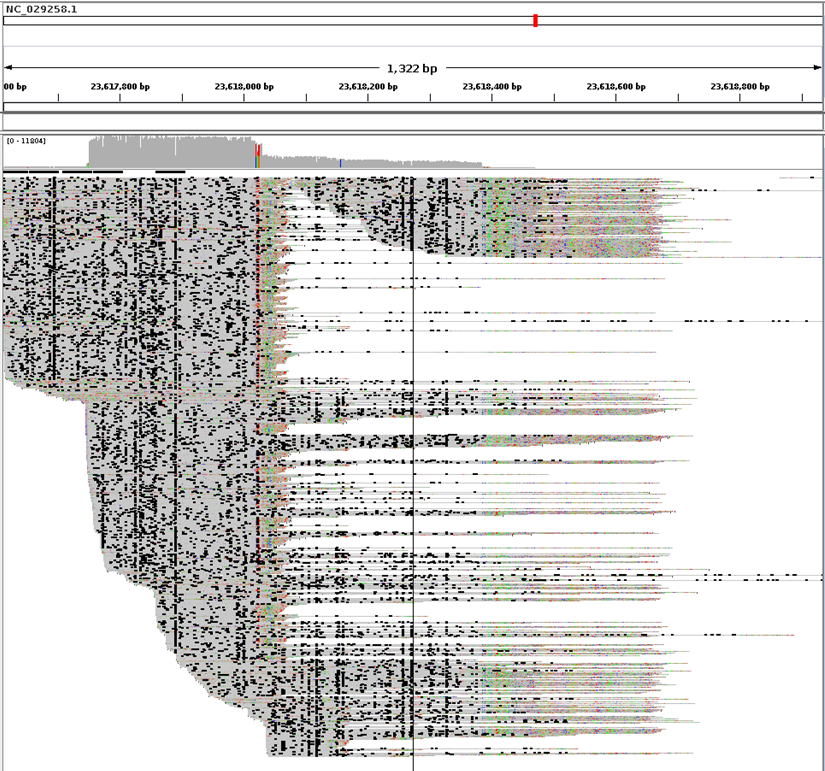 |
| Processed data aligned to the rice chromosome III (aspecific amplification) |
| 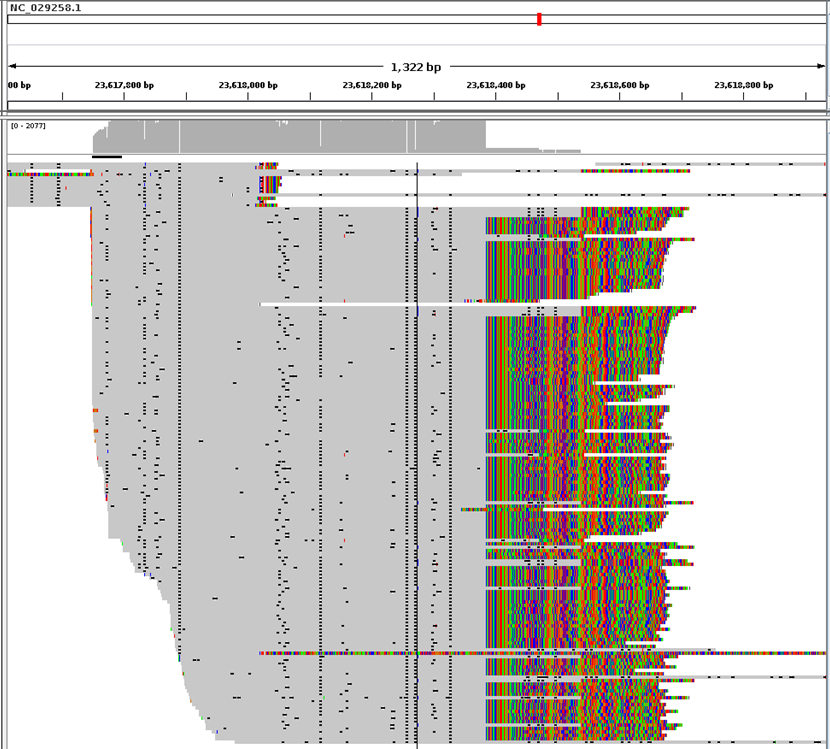 |
| Clustered data aligned to the rice chromosome III (aspecific amplification) |
| 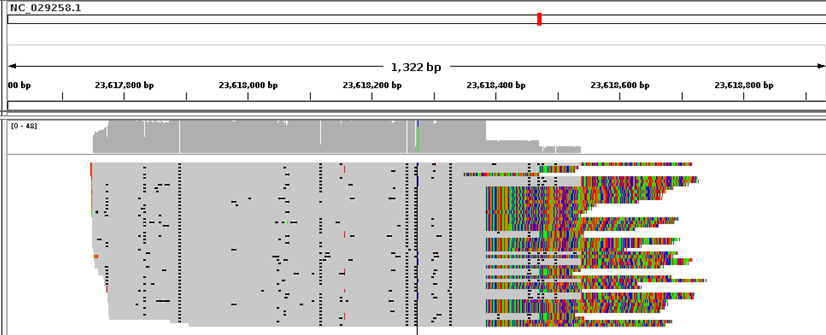 |
| **Additional file 4**: Alignment of the flanking regions to the rice genomic sequences. |
